# Diagnosed hematological malignancies in Bangladesh - a retrospective analysis of over 5000 cases from 10 specialized hospitals

**DOI:** 10.1186/1471-2407-14-438

**Published:** 2014-06-14

**Authors:** Mohammad Sorowar Hossain, Mohd S Iqbal, Mohiuddin Ahmed Khan, Mohammad Golam Rabbani, Hazera Khatun, Sirajam Munira, M Morshed Zaman Miah, Amin Lutful Kabir, Naima Islam, Tashmim Farhana Dipta, Farzana Rahman, Abdul Mottalib, Salma Afrose, Tasneem Ara, Akhil Ranjan Biswas, Mizanur Rahman, AKM Mustafa Abedin, Mahbubur Rahman, ABM Yunus, Louis W Niessen, Tanvira Afroze Sultana

**Affiliations:** 1 BRAC University, Mohakhali, Dhaka, Bangladesh; 2 Centre for Control of Chronic Diseases, icddr’b, Mohakhali, Dhaka, Bangladesh; 3 Dhaka Medical College Hospital, Dhaka, Bangladesh; 4 Chittagong Medical College and Hospital, Chittagong, Bangladesh; 5 Square Hospital Ltd, Dhaka, Bangladesh; 6 Rajshahi Medical College Hospital, Rajshahi, Bangladesh; 7 Delta Medical College and Hospital, Dhaka, Bangladesh; 8 National Institute for Cancer Research and Hospital, Dhaka, Bangladesh; 9 BIRDEM General Hospital, Dhaka, Bangladesh; 10 Bangabandhu Sheikh Mujib Medical University, Dhaka, Bangladesh; 11 Green View Clinic, Dhaka, Bangladesh; 12 Combined Military Hospital, Dhaka, Bangladesh; 13 Centre for Nutrition and Food Security, icddr,b, Mohakhali, Dhaka, Bangladesh; 14 Liverpool School of Tropical Medicine, Liverpool, UK

**Keywords:** AML, CML, ALL, MDS, NHL, HL, MM, Hematological malignancy, Bangladesh

## Abstract

**Background:**

The global burden from cancer is rising, especially as low-income countries like Bangladesh observe rapid aging. So far, there are no comprehensive descriptions reporting diagnosed cancer group that include hematological malignancies in Bangladesh.

**Methods:**

This was a multi-center hospital-based retrospective descriptive study of over 5000 confirmed hematological cancer cases in between January 2008 to December 2012. Morphological typing was carried out using the “French American British” classification system.

**Results:**

A total of 5013 patients aged between 2 to 90 years had been diagnosed with malignant hematological disorders. A 69.2% were males (n = 3468) and 30.8% females (n = 1545), with a male to female ratio of 2.2:1. The overall median age at diagnosis was 42 years. Acute myeloid leukemia was most frequent (28.3%) with a median age of 35 years, followed by chronic myeloid leukemia with 18.2% (median age 40 years), non-Hodgkin lymphoma (16.9%; median age 48 years), acute lymphoblastic leukemia (14.1%; median age 27 years), multiple myeloma (10.5%; median age 55 years), myelodysplastic syndromes (4.5%; median age 57 years) and Hodgkin’s lymphoma (3.9%; median age 36 years). The least common was chronic lymphocytic leukemia (3.7%; median age 60 years). Below the age of 20 years, acute lymphoblastic leukemia was predominant (37.3%), followed by acute myeloid leukemia (34%). Chronic lymphocytic leukemia and multiple myeloma had mostly occurred among older patients, aged 50-over.

**Conclusions:**

For the first time, our study presents the pattern and distribution of diagnosed hematological cancers in Bangladesh. It shows differences in population distributions as compared to other settings with possibly a lower presence of non-Hodgkin lymphoma. There might be under-reporting of affected women. Further studies are necessary on the epidemiology, genetics and potential environmental risk factors within this rapidly aging country.

## Background

Once considered a problem exclusive to Western countries, cancer is becoming a leading cause of death and disability in low-income countries. In 2012 more than 57% of the 14.1 million cancer cases and 65% of the 8.2 million cancer deaths occurred in these settings [1]. Even if current global cancer rates would remain unchanged, low- and lower-middle income countries would bear the burden of more than two thirds of the projected 21.7% million new cancer cases and 13 million cancer deaths by 2030 [1]. Despite current or future efforts, the challenges of tackling cancer are enormous, since the occurrence of cancer in these settings will continue to rise due to increasing in lifespans through better control of communicable diseases [2].

Bangladesh shows major advances in relation to the management of infectious diseases as recently highlighted in *Lancet*[3], the chronic diseases in particular cancers are less prioritized [4]. The status of cancer in this country is largely unknown, as there is no population-based cancer registry nor a national cancer registry of any other kind. According to WHO, Bangladesh is experiencing increasing cancer burden with estimated 122,715 new cancer cases in 2012 [1] The number of new cases is projected to increase approximately by 77% in 2030. These WHO estimates may not reflect the real cancer status, as the estimates were extrapolated based on the incidence and mortality rates from regional data (India) and a single hospital [1]. The figures are likely to underestimated as many cases go unreported due to lack of awareness, education, misconceptions, and poverty among populations, in addition due to poor health systems and poor governance [5]. Moreover, overall cancer care and management systems are below international standard due to the high treatment costs, the lack of oncologists and insufficient infrastructure.

Hematological malignancies (HM) comprise approximately 6.5% of all cancer incidences worldwide in 2012 [1]. Although prevalence of these malignancies are much lower in Asia and Africa then in Western countries, the incidence of these malignancies is drastically increasing in low-income settings, while these increasing trends are not observed in Western countries [6-8]. WHO predicts that the number of blood-related cancer cases would increase about 48% in less developed countries by 2030 as compared to 2012 [1].

HM are primary cancers originating from cells of the bone marrow and lymphatic system [9] and there are three main categories including forms of leukemia, lymphoma, and multiple myeloma (MM). Other includes myelodysplastic syndrome (MDS), polycythaemia vera, and primary myelofibrosis. The four common types of leukemia are: acute myeloid leukemia (AML), acute lymphoblastic leukemia (ALL), chronic myeloid leukemia (CML), chronic lymphocytic leukemia (CLL). The two main types of lymphoma are: non-Hodgkin lymphoma (NHL) and Hodgkin’s lymphoma (HL). The causes of hematological malignancies remain unclear, but these are believed to be linked with environmental exposure of chemicals (such as pesticides, benzene, smoking etc.), as well as ionizing radiation and infectious agents. The incidence of HM varies with geography, age and race/ethnicity, suggesting different etiological factors may contribute for the development of these malignancies [10,11].

Information pertaining to the epidemiological aspects of HM in South Asian population is limited [12]. In case of Bangladesh, there is no published report on HM prevalence and treatment. An understanding of the epidemiological aspects of HM would surely contribute to identify the risk factors in our environmental background and would provide epidemiological basis for devising the cancer care management and preventive strategies of these malignancies. In the present retrospective study, we report for the first time the overall pattern and age distribution of hematological malignancies in Bangladesh.

## Methods

Bangladesh has one state-run specialized cancer hospital, fourteen oncology units of public medical teaching hospitals and few private clinics and hospitals available for the management of all cancer patients. While over 70% of the population live in rural areas, most of the tertiary health care facilities for cancer treatment are centred in the capital city, Dhaka [13]. Therefore, patients seeking for cancer treatment travel to Dhaka. In our retrospective analysis, the study population consists of all patients with hematological malignancies diagnosed at the tertiary and specialized hospitals in Dhaka from January 2008 to December 2012. Not all hospitals provided data over the above-mentioned time period since some of them had not started to handle HM cases. Data extraction occurred for those hospitals that have been capable of making a definite diagnosis of hematological malignancies. Two hospitals outside of Dhaka city - Chittagong Medical College and Hospital and Rajshahi Medical College Hospital were included (Table 1).

**Table 1:** Case distribution by institution and year

**Institution**	**Duration (year)**	**No of cases (n)**	**Existing facilities for HM diagnosis**
CMCH	2011-12	153	Morphological
CMH	2012	75	Morphological, cytochemistry, karyotyping, immunocytochemistry
RMCH	2012	148	Morphological
Green View Hospital	2012	264	Morphological, cytochemistry
Delta Hospital	2008-12	146	Morphological, cytochemistry, immunocytochemistry
Square Hospital Ltd.	2008-12	180	Morphological, immunocytochemistry
NICRH	2010-12	781	Morphological
BIRDEM	2008-12	208	Morphological, karyotyping
BSMMU	2008-12	1907	Morphological, cytochemistry, immunocytochemistry
DMCH	2008-12	1151	Morphological
Total	2008-12	5013	

Our study is based on the recorded diagnoses of HM in the various participating centres. The source of the information included bone marrow morphology databases from the hematopathology laboratories as well as inpatient and outpatient case registers maintained at the participating hospitals. The participating hospital mostly provided both pathological diagnoses and clinical management information. The exception was BIRDEM General Hospital where service is limited to diagnostic services. Diagnoses were carried out on the basis of clinical features, blood counts, peripheral blood films and bone marrow morphology including cytochemical staining and immunocytochemistry where available. Facilities available for the diagnosis of HM at various participating centres are shown in Table 1. Morphological typing of malignancies was carried out according to “French American British (FAB)” classification system. Although WHO classification (2008) for haematological malignancies is based mostly on immunophenotype and molecular markers, we had to stick to FAB classification because we had limited facilities for immunophenotyping or cytogenetic studies. Our diagnoses were supplemented by criteria from the 2008 WHO classification whenever facilities were available to fulfil the criteria as shown in Table 1.

As there is no proper and effective hospital record keeping system in Bangladesh, there are always possibilities that the same patient might visited multiple times in the same hospitals and/or different hospitals. These would eventually undermine the actual diagnosed cases of HM. To eliminate these possibilities, raw data were painstakingly analyzed to clean up such duplicated, re-enrolled cases. Individual co-authors initially sorted and cleaned up the data of their own hospitals. Later, a team of investigators including MSH, MSI and TAS carefully cross-checked all data from participating centres for potential duplicates or re-enrolled cases depending on various parameters including name of the patient, age, year of diagnosis and the name of the hospital attended. The initial dataset contained 5338 cases. At the end, 5013 diagnosed cases were analyzed in our study after removing all duplicated or re-enrolled cases (6% of all cases). It was not possible to get information on the cancer-associated deaths due to lack of proper record keeping system. Final data representing individual cases were analyzed by SPSS version 17 statistical software. The study protocol was ethically approved by the Ethical Review Committee of Diabetic Association of Bangladesh under the official memo no. BADAS-ERC/EC/13/0081.

## Results

A total of 5013 diagnosed hematological malignancy-related cases extracted from ten different tertiary hospitals were retrospectively analysed in this study. Of these, patients aged between 2 to 90 years, 3468 were males (69.2%) and 1545 females (30.8%), with a male to female ratio of 2.2:1 (Table 2). Male-biased prevalence of HM was statistically significant (*χ*^2^ = 54.28, *P* = 0.001). Among all hematological malignancies cases, 6.7% (n = 338) were children aged under 20 years old (Table 3). The combined median age at diagnosis for all hematological malignancies was 42 years.

**Table 2:** Distribution pattern, median age at diagnosis and male–female ratio of hematological malignancies in Bangladesh

**HM types**	**Cases**	**Distribution (%)**	**Median age (year)**	**Male–female ratio**
**AML**	1417	28.3	35	1.9
**CML**	912	18.2	40	2.1
**ALL**	706	14.1	27	2.1
**CLL**	183	3.7	60	2.9
**NHL**	846	16.9	48	3.6
**HL**	196	3.9	36	3.4
**MM**	528	10.5	55	2.1
**MDS**	225	4.5	57	1.9
Total	5013	100	42	2.2

**Table 3:** Age-group specific distribution of hematological malignancies in Bangladesh

**Age group**	**AML**	**CML**	**ALL**	**CLL**	**NHL**	**HL**	**MM**	**MDS**	**Total cases**	**Age-specific**
	**% (n)**	**% (n)**	**% (n)**	**% (n)**	**% (n)**	**% (n)**	**% (n)**	**% (n)**	**n**	**%**
**Under 20**	8.1 (115)	4.1 (44)	17.8 (126)	1.6 (3)	2.1 (18)	14.3 (28)	0.2 (1)	1.3 (3)	338	6.7
**20-29**	24.5 (347)	21.3 (194)	38.8 (274)	1.6 (3)	17.6 (149)	21.9 (43)	1.1 (6)	6.2 (14)	1030	20.5
**30-39**	22 (312)	23.8 (217)	17 (120)	4.4 (8)	12.5 (106)	15.8 (31)	2.8 (15)	5.8 (13)	822	16.4
**40-49**	18.1 (256)	21.1 (192)	10.6 (75)	9.3 (17)	20.6 (174)	25 (49)	19.1 (101)	9.8 (22)	886	17.7
**50-59**	14.1 (200)	16.4 (150)	6.9 (49)	20.8 (38)	24.1 (204)	15.8 (31)	36.6 (193)	29.3 (66)	931	18.6
**60-69**	8.1 (115)	10.3 (94)	5.8 (41)	28.4 (52)	16.4 (139)	4.1 (8)	24.8 (131)	25.8 (58)	638	12.7
**70+**	5.1 (72)	2.3 (21)	3 (21)	33.9 (62)	6.6 (56)	3.1 (6)	15.3 (81)	21.8 (49)	368	7.3
**Total cases**	1417	912	706	183	846	196	528	225	5013	100

AML was the most frequent HM (28.3%) in Bangladesh with a median age of 35 years. This was followed by CML (18.2%, median age 40 years), NHL (16.9%, median age 48 years), ALL (14.1%, median age 27 years), MM (10.5%, median age 55 years), MDS (4.5%, median age 57 years) and HL (3.9%, median age 36 years). The least common HM was CLL (3.7%, median age median age 60 years) [Table 2].

Moreover, sex-specific analysis was also performed for the overall and individual cancer cases. No significant difference was observed in the median age of presentation (male 42 years; female 41 years) for both sexes except for ALL (male 25 years, female 30 years), HL (male 40 years, female 28 years), CLL (male 61 years, female 50 years) and MDS (male 59.5 years and female 55 years).

Age-group specific distribution showed that three types of HM including AML (64.6%), CML (66.2%), ALL (66.4%), and HL (62.7%) were predominantly observed in the young adults aged 20-49 years (Table 3). Interestingly, four other HM were mostly occurred among older patients aged 50-over 70 years. These include CLL (83.1%), MM (76.7%) and MDS (76.9%). On the other hand, NHL was almost evenly distributed among 20–49 years (50.7%) and 50-over 70 years (49.3%) age group (Table 3).

Among 338 childhood HM cases analyzed in our study, 257 cases were in 15–19 year age group, while 81 cases were in 0–14 year age group. In children younger than 20 years, ALL (37.3%) was the predominant type of HM, followed by AML (34%), CML (13%), HL (8.3%) and NHL (5.3%) [Table 3].

## Discussion

To our knowledge, this is the first comprehensive report on the burden of hematological malignancies in Bangladesh. In contrast to the WHO estimates, our multi-centred hospital-based data present a different picture: the leukemias constituted approximately two thirds of (64.3%) all HM cases, while NHL accounted for 16.9%, followed by MM (10.5%) and HL (3.9%) [Table 2]. A similar pattern of leukemias (Age-standardized incidence rate or ASR per 100,000 is 3.3), NHL (ASR per 100,000 is 3), and multiple myeloma is observed in India. The WHO prediction, the commonest type of HM was NHL (ASR is 1.9 per 100,000 persons), which was followed by leukemias (ASR 1.7 per 100,000 persons), HL and multiple myeloma [1]. In Pakistan, NHL is the most prevalent type of HM [1]. In US, NHL is the commonest cancer among HM, which is 1.5 times that of all leukemias [14]. In contrast, all leukemia cases were over three times higher as compared to NHL cases in Bangladesh. In other Asian countries including Japan, Korea and Singapore, NHL is the most frequent hematological malignancies [1,7]. In our study, this unexpected discrepancy might be due to lack of proper referral system in some participating centres. Although lymphoma is a haematological disorder, a small number of patients might have been admitted to the medical oncology department. Moreover, year-specific data was not available for four of the participating centres (Table 1). Yet, our hospital-based study represents the overall current country picture on HM. Additional population-based studies are warranted to reveal the true incidence of HM.

Unlike Western countries, the hematological malignancies in Bangladesh seem to afflict younger population as is indicated by the overall median age at diagnosis was 42 years. Perhaps the true median age may be even lower, given the under-representation of children in this study. Acute leukemias (AML and ALL), CML and lymphoma (NHL and HL) were found to occur in relatively young adults with a median age ranging from 27 to 48 years (Table 4). On the other hand, MM, CLL and MDS were associated with relatively older individuals (median age at diagnosis was 55 to 60 years). It is generally argued that young age phenomenon of cancers might be due to the lower life expectancy and younger population structure of a respective country [12,15,16]. In Bangladesh, 33% of the population is under 15 years old and the median age of the entire population is 23.4 years. Another often cited reason is related to the underreporting cases of older individuals possibly because of several socioeconomic and cultural reasons. However, the lower life expectancy explanation is doubtful as in economically developed Singapore, Hong Kong, South Korea, Taiwan and Japan - with much higher life expectancies similar to those seen in Western countries - are also affected by CML [17], NHL [18] and MDS [19] at relatively young ages. The reasons behind this phenomenon are unclear. However, it is likely that the multiple factors including genetic, infections and other environmental factors might play crucial role in this young age phenomenon in Asia. Moreover, an analysis with migrant Bangladesh/Indian living in economically developed regions with different life expectancy may help to understand this issue further*.*

**Table 4:** Median age at diagnosis of hematological malignancies in Bangladesh as compared to India and US

**HM**	**Median age at diagnosis (year)**		
	**Bangladesh**	**India**	**US **[23]
**AML**	35	30	67
**CML**	40	38-40	64
**ALL**	27	23	14
**CLL**	60	60	71
**NHL**	48	49.5*	66
**HL**	36	31.9*	38
**MM**	55	55-56	69
**MDS**	57	46.1*	60-70
**All types**	42	~ 40**	65-70

*Mean age.

**Estimated, specific reference is not available.

Our retrospective analysis included 6.7% childhood (under 20 years) HM cases (n = 338), of which approximately 76% cases comprised of adolescent (15–19 years age group) cancer patients. It is important to mention that childhood cases were under represented in the present study as the information was obtained from the hematology department of some of the major participating centres which usually manage childhood (0–14 years) cases in separate facilities. Despite this limitation, we can present an overview of children hematological malignancies in Bangladesh. Leukemias were found to be the most common blood associated cancers, constituting of 92.6% of all children HM cases (Table 3). Among these leukemia patients, ALL is the most frequent cancer accounting for 37.3% (n = 126) followed by AML (34%, n = 115) and CML (13%, n = 44). Lymphoma accounted for 13.6% (HL-8.3% and NHL-5.3%) of all children cases. Like Western countries and Bangladesh, the leukemias were the most prevalent childhood cancers in India with a relative proportion ranging from 25-40% of all childhood cancers. Approximately 60 to 85% of all reported leukemias was ALL [20]. Further extensive study is necessary to understand the pattern and distribution of HM among children in the country.

Studies all over the world have revealed that hematological malignancies is gender-skewed, often affecting men more than women. We also found that men were more involved than women, with an overall male to female ratio of 2.2. For lymphoma, this ratio (~3.4) seems to be much higher than normal trend. Probably, female cases were underreported considering the socioeconomic status of the families and in low-resource settings usually men often get priority while seeking medical attention. The higher prevalence of HM in males might be the result of increased exposure to environmental and occupational risk factors, smoking, alcohol consumption as well as different hormonal and genetic background of males and females [10,21-23].

Acute leukemias including AML and ALL are the most prevalent HM affecting Bangladeshi population, accounting for 42.4% (n = 5013) of all HM cases, while these two constituted 66% (n = 3218) of leukemia cases (Table 2). The frequency of AML is two times higher than that of ALL in Bangladesh. The incidence of AML is relatively common in North America, Europe, and Oceania, while adult AML is rare in Asia and Latin America [24]. AML generally affects older individuals with a median age at presentation of around 65 years in Western countries [11] and it accounts for ~ 29% of all leukemias in adults in US. Our study showed that acute leukemias tend to affect relatively young adults aged 20–49 years (66.4% cases) [Table 3]. The median age at onset for AML (35 years) in Bangladesh is higher than in India (30 years) [12]. Apart from lower life expectancy prevailing in the Indian subcontinent, it is likely that elderly patients may not be reporting to the hospitals because of relatively rapid progression of AML. ALL occurs in people of all ages but it exhibits bimodal age-specific curve with peaks in youngest (<20 years) and oldest ages (>50 years). In our study we observed that more than 55% of the ALL occurs in young adults (20–40 years) with a median of 27 years (Table 3). In US, the overall median age for ALL is only 14 years, since approximately 60% of the cases occur in children under 20 years old [25]. In case of adult, the median age was 38 years [26].

Chronic leukemias constituted 21.9% of all HM and 34% of leukemias in Bangladesh. CML is the second most common type (18.2%, n = 912), while CLL is the least frequent (3.7%, n = 183) HM (Table 2). The frequency of CML is five times higher than CLL. The pattern of CML occurrence is different in India and Africa where it is the most common form of leukemia [12,27]. In Sudan, the incidence of CML is very high, being the predominant cancer in men in last 25 years [28]. CLL is a rare hematological malignancy in Asia while this is the commonest form of leukemia affecting elderly in Western countries with a median age of 70 years [11] . In US, for instance, CLL constitutes about ~34% of all leukemia [14]. In Bangladesh and India, CLL is found to occur mainly in adults (median age ~60 years) [29]. The lower frequency of CLL in Bangladesh and its neighbouring countries may not be associated with lower life expectancy since the incidence of CLL in Japan is at least 4 to 5 times lower than Western countries; despite Japanese have the highest life expectancy in the world [30]. It is possible that genetics and environment may play important role in its development [11,31].

Malignant lymphoma constituted 20.8% of all HMs in our study (Table 2). Out of 1052 lymphoma cases, NHL accounted for 80.4% while 19.6% was for HL. The frequency of NHL and HL observed in the present study was almost similar to the earlier report from India [32]. NHL is one of the commonest cancers in developed countries, but the incidence is relatively lower in Asia [18]. This is the most frequent HM of many Asian [1,18] and African countries [33]. In Bangladesh, it constituted 16.9% of all HM with a median age of 48 years. On the other hand, HL is the most common cancer of young adults in developed countries. The age distribution of HL is bimodal, the first being young adults (age 15–35 years) and the second being those in older individuals over 55 years old. In our study, 62.7% of all HL cases occurred among young adults (<50 years) with a median of 36 years. The similar frequency of HL pattern was also observed in India where mean age of diagnosis was 31.9 years [32].

In our study, nearly 76.7% of the MM patients aged over 50 years. This accounted for 10.5% of all HM. In Western countries, the median age at diagnosis is 65–70 years, which is significantly higher as compared to Asian countries like India, Japan and China [34]. The median age at onset in Bangladesh is 55 years, which similar to that in India (55–56 years). The incidence varies among different races or geographic location. For instance, the higher incidence has been reported for African Americans as compared to Caucasians [35].

Myelodysplastic syndromes (MDS) are relatively a condition affecting elderly. It can transform to AML. The incidence of MDS is unclear worldwide because of historical lack of population-based registration. In Bangladesh, MDS constituted approximately 4.5% of HM and 76.9% (n = 225) of the patients were over 50 years old with a median age of 57 years, which is similar to that reported in Japan [19]. In case of India, the MDS patients were much younger with a mean age of 46.1 years [36], while Western countries have median age ranges from 60–70 years [37].

Large sample size (over 5000 diagnosed cases) is the strength of our study. However, in our retrospective study, we could not provide any information on the mortality and survivorship of HM, since there is no proper follow-up system to track terminally-ill patients in Bangladeshi hospitals. Detailed clinical data were also not available because of incomplete record keeping system. Like other low-income countries, many of these limitations are intrinsic to Bangladesh health system. Time-trend analysis was not also possible due to lack of homogeneity of data obtained from different hospitals, which has been described in the method section and elsewhere.

## Conclusion

This study of a large number of HM patients is a very first step in understanding the patterns and distribution of HM in Bangladesh. Further investigations are necessary to understand the epidemiology, potential risk factors, biology and genetics of hematological malignancies in this country in rapid transition.

## Abbreviations

HM: Hematological malignancies; AML: Acute myeloid leukemia; ALL: Acute lymphoblastic leukemia; CML: Chronic myeloid leukemia; CLL: Chronic lymphocytic leukemia; NHL: Non-Hodgkin lymphoma; HL: Hodgkin’s lymphoma; MM: Multiple myeloma; MDS: Myelodysplastic syndromes.

## Competing interests

The authors declare that they have no competing interests.

## Authors’ contributions

MSH, MSI, LWN and TAS conceived of the study, carried out the analysis, contributed to the interpretation of the data and the writing of the manuscript. MSH drafted the initial manuscript. MGR, HK, SM, MMZM, ALK, NI, TFD, FR, JF, AM, SA, TA, ARB, MR, MSH, MSI, TAS, MR, ABMY and MAK contributed to the data acquisition. MSH, MSI, TAS and LWN critically revised the manuscript. All authors read and approved the final manuscript.

## Pre-publication history

The pre-publication history for this paper can be accessed here:

http://www.biomedcentral.com/1471-2407/14/438/prepub
